# L-cystathionine inhibits oxidized low density lipoprotein-induced THP-1-derived macrophage inflammatory cytokine monocyte chemoattractant protein-1 generation via the NF-κB pathway

**DOI:** 10.1038/srep10453

**Published:** 2015-05-28

**Authors:** Mingzhu Zhu, Junbao Du, Angie Dong Liu, Lukas Holmberg, Selena Y Chen, Dingfang Bu, Chaoshu Tang, Hongfang Jin

**Affiliations:** 1 Department of Pediatrics, Peking University First Hospital, Beijing 100034, P. R. China; 2 Key Laboratory of Molecular Cardiology, Ministry of Education, Beijing 100191, P. R. China; 3 Center for Molecular and Translational Medicine, Peking University Health Science Centre, Beijing 100191, P. R. China; 4 Department of Medical and Health Sciences, Linköping University, Linköping 58183, Sweden; 5 University of California, San Diego, La Jolla, California, 92093, United States of America; 6 Central Laboratory, Peking University First Hospital, Beijing 100034, P. R. China; 7 Department of Physiology and Pathophysiology, Peking University Health Science Centre, Beijing 100191, P. R. China

## Abstract

This study aimed to explore whether and how L-cystathionine had any regulatory effect on the inflammatory response in THP-1-derived macrophages cultured *in vitro* under oxidized low-density lipoprotein (ox-LDL) stimulation. The human monocyte line THP-1 cell was cultured *in vitro* and differentiated into macrophages after 24 hours of PMA induction. Macrophages were pretreated with L-cystathionine and then treated with ox-LDL. The results showed that compared with the controls, ox-LDL stimulation significantly upregulated the expression of THP-1-derived macrophage MCP-1 by enhancing NF-κB p65 phosphorylation, nuclear translocation and DNA binding with the MCP-1 promoter. Compared with the ox-LDL group, 0.3 mmol/L and 1.0 mmol/L L-cystathionine significantly inhibited the expression of THP-1-derived macrophage MCP-1. Mechanistically, 0.3 mmol/L and 1.0 mmol/L L-cystathionine suppressed phosphorylation and nuclear translocation of the NF-κB p65 protein, as well as the DNA binding activity and DNA binding level of NF-κB with the MCP-1 promoter, which resulted in a reduced THP-1-derived macrophage MCP-1 generation. This study suggests that L-cystathionine could inhibit the expression of MCP-1 in THP-1-derived macrophages induced by ox-LDL via inhibition of NF-κB p65 phosphorylation, nuclear translocation, and binding of the MCP-1 promoter sequence after entry into the nucleus.

L-Cystathionine is an important intermediate product in the sulfur-containing amino acid metabolism system. It is now well established that L-cystathionine is produced during the metabolic transformation process of methionine to cysteine^1^. In recent years, researchers have found that certain sulfur-containing amino acids including methionine, cystine, cysteine, taurine, hydrogen sulfide (H_2_S) and sulfur dioxide play an important biological role in the regulation of cardiovascular function. Moreover, L-cystathionine plays a central role in the sulfur transferring process, but there is little known about its relatively independent biological effects^2^,^3^. Preliminary studies suggested that L-cystathionine was involved in eliminating superoxide radicals^4^,^5^, apoptosis^6^, liver protection^7^,^8^ and endoplasmic reticulum stress^9^, etc. Inflammation is a defensive reaction of living tissue^10^,^11^ and a very common and important basic pathological process. For example, it affects the development of atherosclerosis^12^,^13^. Mononuclear macrophages are critical in vascular inflammation. MCP-1 released from mononuclear macrophages has a strong chemotaxis towards monocytes in the blood, inducing the expression of adhesion molecules by monocytes and endothelial cells mediating the adhesion and migration of various inflammatory cells, especially inflammatory cells, into the injured endangium, and regulating the vascular inflammation.

It is still unclear to date whether L-cystathionine has any biological role in inflammatory regulation in macrophages. Therefore, the study was undertaken to examine if L-cystathionine in macrophages impacts inflammatory cytokine MCP-1 expression in THP-1-derived macrophages induced by ox-LDL, and its possible mechanisms.

## Results

### Ox-LDL induced the expression of the THP-1-derived macrophage MCP-1

**Figure 1:**
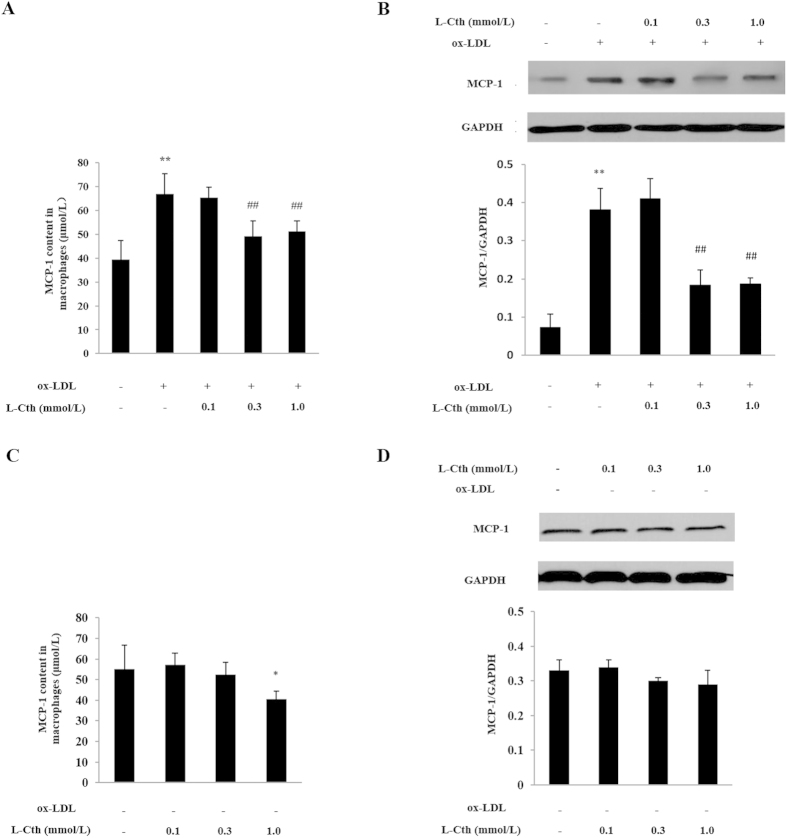
L-cystathionine inhibited the expression of MCP-1 induced by ox-LDL in the THP-1-derived macrophage. The cells were pretreated with L-cystathionine (0.1 mmol/L, 0.3 mmol/L or 1.0 mmol/L, respectively) for 30 min, and were then stimulated with 50 mg/L ox-LDL for 24 h. (**A**) The content of MCP-1 in the supernatant of THP-1-derived macrophage detected by ELISA. (**B**) The expression of MCP-1 protein in the THP-1-derived macrophage detected by western blot. (**C**, **D**) The basal expression of MCP-1 without ox-LDL incubation detected by ELISA and western blot. L-Cth: L-cystathionine. **P < 0.01 compared with control group, ##p < 0.01 compared with ox-LDL group. Data are presented as mean ± SD (n = 3) of three independent experiments performed in triplicate.

Firstly, enzyme-linked immunosorbent assay (ELISA) and Western blotting methods were used to detect the expression levels of MCP-1 in THP-1-derived macrophage. The results showed that the expression levels of MCP-1 increased significantly after treating cells with 50 mg/L ox-LDL compared with control group without ox-LDL treatment (Fig. 1A, Fig. 1B).

### L-cystathionine inhibited the ox-LDL-stimulated MCP-1 expression in the THP-1 derived macrophages

For the purpose of exploring the impact of L-cystathionine on expression of THP-1-derived macrophage-derived MCP-1 stimulated by ox-LDL, firstly THP-1 derived macrophages were pretreated with L-cystathionine for 30 minutes. They were then stimulated with 50 mg/L of ox-LDL for 24 hours. ELISA was used to detect the expression levels of MCP-1 in the supernatant of the THP-1-derived macrophage. The results showed that in comparison to the control group, the expression levels of MCP-1 in cell supernatant in the ox-LDL group were significantly increased. However, pretreatment of L-cystathionine dose-dependently declined the expression levels of MCP-1 (Fig. 1A). A subsequent western blot test showed that ox-LDL significantly stimulated the expression of MCP-1 protein in THP-1-derived macrophages. However, L-cystathionine dose-dependently inhibited the expression of THP-1-derived macrophage MCP-1 induced by ox-LDL (Fig. 1B). Both ELISA and Western blot results showed that L-cystathionine had no effect on the expression of MCP-1 without ox-LDL stimulation (Fig. 1C, Fig. 1D).

### L-cystathionine inhibited the inflammatory response of THP-1-derived macrophages induced by ox-LDL via the NF-κB pathway

Through the aforementioned study, we found that L-cystathionine could inhibit the expression of inflammatory cytokine MCP-1 in a THP-1-derived macrophage induced by ox-LDL, though its regulatory mechanism remains unclear. The NF-κB signaling pathway is a core part of the regulation of a cellular inflammatory response, and p65 is a major subunit of the NF-κB signal transduction pathway. In this section, we examined if and how the regulating effect of L-cystathionine on the inflammatory response of THP-1-derived macrophages was mediated by the NF-κB signaling pathway by detecting the phosphorylation levels of NF-κB p65, the nuclear translocation of NF-κB p65, as well as DNA binding activity of NF-κB and the DNA binding level of NF-κB with the MCP-1 promoter.

**Figure 2:**
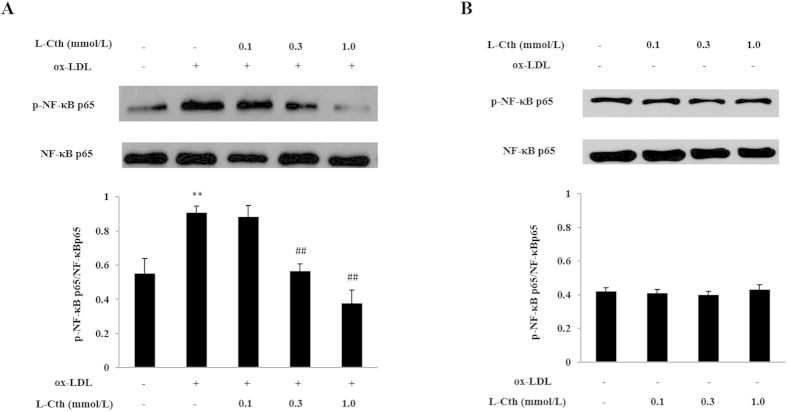
L-cystathionine inhibited the phosphorylation of NF-κB p65 protein induced by ox-LDL in the THP-1-derived macrophage. The cells were pretreated with L-cystathionine (0.1 mmol/L, 0.3 mmol/L or 1.0 mmol/L, respectively) for 30 min, and were then stimulated with 50 mg/L ox-LDL for 30 min. (**A**) The effect of L-cystathionine on phosphorylation level of NF-κB p65 protein induced by ox-LDL in the THP-1-derived macrophage detected by western blot. (**B**) The basal phosphorylation level of NF-κB p65 protein in the THP-1-derived macrophage without ox-LDL treatment detected by western blot. L-Cth: L-cystathionine. **P < 0.01 compared with control group, ^##^p < 0.01 compared with ox-LDL group. Data are presented as mean ± SD (n = 3) of three independent experiments performed in triplicate.

To begin, we studied whether phosphorylation levels of NF-κB p65 was involved in MCP-1 expression by L-cystathionine. A western blot test was used to detect the phosphorylation levels of NF-κB p65. THP-1-derived macrophages were pretreated with L-cystathionine for 30 min, and then were stimulated with 50 mg/L ox-LDL for 30 min. The results show that ox-LDL significantly enhanced phosphorylation levels of the THP-1-derived macrophage NF-κB p65 protein. Meanwhile, L-cystathionine dose-dependently declined phosphorylation levels of NF-κB p65 protein (Fig. 2A). However, L-cystathionine had no effect on the phosphorylation levels of the NF-κB p65 protein without ox-LDL stimulation (Fig. 2B).

**Figure 3:**
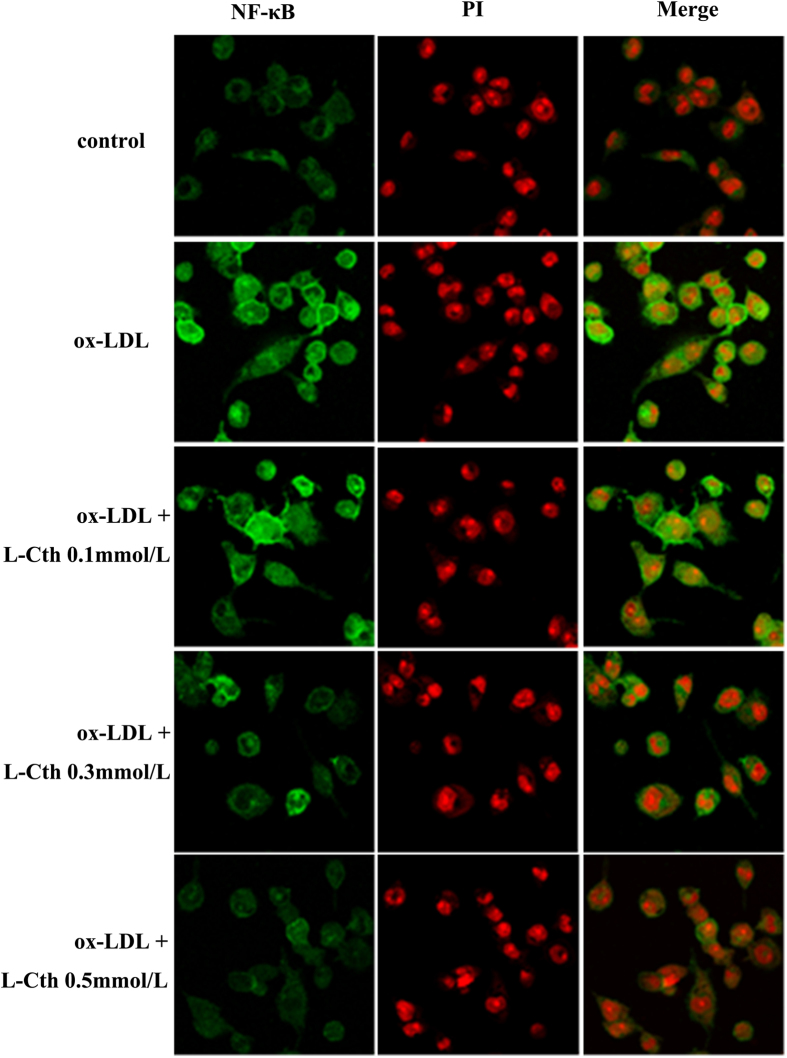
L-cystathionine prevented nuclear translocation of NF-κB p65 induced by ox-LDL in the THP-1-derived macrophage. The effect of L-cystathionine on nuclear translocation of NF-κB p65 induced by ox-LDL in the THP-1-derived macrophage observed by immunofluorescence assay. Red color indicates the nucleus, and green color indicates the expression of NF-κB p65. Data are from three independent experiments performed in triplicate. L-Cth: L-cystathionine.

We then used immunofluorescence methods to observe the effect of L-cystathionine on nuclear translocation of p65 in the THP-1-derived macrophage. THP-1-derived macrophages were pretreated with L-cystathionine for 30 min, and then were stimulated with 50 mg/L ox-LDL for 30 min. The results demonstrated that ox-LDL significantly enhanced nuclear translocation of the THP-1-derived macrophage NF-κB p65. Meanwhile, it was dose-dependently inhibited when the cells were pretreated with L-cystathionine (Fig. 3).

**Figure 4:**
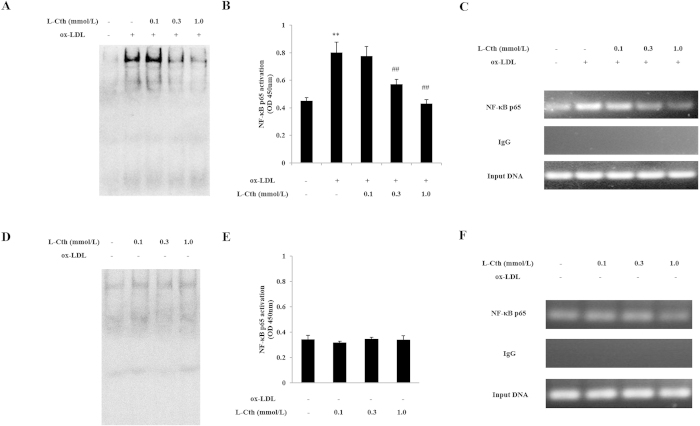
L-cystathionine decreased DNA binding activity of NF-κB p65 induced by ox-LDL in the THP-1-derived macrophage. The cells were pretreated with L-cystathionine (0.1 mmol/L, 0.3 mmol/L or 1.0 mmol/L, respectively) for 30 min, and were then stimulated with 50 mg/L ox-LDL for 30 min. (**A**) The effect of L-cystathionine on DNA binding activity of NF-κB p65 induced by ox-LDL in the THP-1-derived macrophage detected by EMSA. (**B**) The effect of L-cystathionine on DNA binding activity of NF-κB p65 induced by ox-LDL in the THP-1-derived macrophage detected by ELISA. (**C**) The effect of L-cystathionine on DNA binding level of NF-κB p65 with MCP-1 promoter induced by ox-LDL in the THP-1-derived macrophage measured by ChIP. (**D**, **E**, **F**) The basal DNA binding activity of NF-κB p65 in the THP-1-derived macrophage without ox-LDL treatment. L-Cth: L-cystathionine. **P < 0.01 compared with control group, ^##^p < 0.01 compared with ox-LDL group. Data are presented as mean ± SD (n = 3) of three independent experiments performed in triplicate.

To study whether L-cystathionine has an inhibitory effect on the DNA binding activity of NF-κB, we used electrophoretic mobility shift assay (EMSA) to measure the effect of L-cystathionine on the DNA binding activity of p65 in the THP-1-derived macrophage. THP-1- derived macrophages were pretreated with L-cystathionine for 30 min, and then were stimulated with 50 mg/L ox-LDL for 30 min. The results indicated that ox-LDL significantly increased the DNA binding activity of p65 in the THP-1-derived macrophage. However, DNA binding activity of NF-κB p65 in the THP-1-derived macrophage was dose-dependently inhibited when the cells were pretreated with L-cystathionine (Fig. 4A). Moreover, we also used the ELISA method to measure the DNA binding activity of NF-κB p65. The results showed that ox-LDL markedly enhanced the DNA binding activity of p65 in the THP-1-derived macrophage. DNA binding activity of NF-κB p65 was dose-dependently inhibited in the cells pretreated with L-cystathionine (Fig. 4B). However, both EMSA and ELISA results showed that L-cystathionine had no effect on the DNA binding activity of p65 without ox-LDL stimulation (Fig. 4D, Fig. 4E).

Finally, we used chromatin immunoprecipitation (ChIP) method to measure the effect of L-cystathionine on binding levels of p65 with the MCP-1 promoter. Similarly, THP-1-derived macrophages were firstly pretreated with L-cystathionine for 30 mins, and then were stimulated with 50 mg/L ox-LDL for 30 mins. We found that ox-LDL significantly increased the DNA binding level of p65 with the MCP-1 promoter in the THP-1-derived macrophage. However, the DNA binding level of p65 with the MCP-1 promoter induced by ox-LDL was dose-dependently reduced when pretreating with L-cystathionine, respectively (Fig. 4C). L-cystathionine had no impact on the DNA binding level of p65 without ox-LDL stimulation (Fig. 4F).

## Discussion

L-cystathionine plays a key role in sulfur-containing amino acid metabolism in mammals. Cysthathionine-β-synthase (CBS) is a fundamental enzyme in L-cystathionine synthesis, which catalyzes the condensation of serine or cysteine with homocysteine to form L-cystathionine and water or L-cystathionine and H2S. In the 1999 study on change in CBS gene expression during the process of THP-1-derived embryonic development, Quéré, *et al.* found that CBS was expressed in a variety of tissues during early embryonic development. The highest levels of CBS expression were in nerve and heart tissue, while in adults CBS was only expressed in the liver and the brain^14^. However, in 2003 Robert, *et al.* proved that CBS transcription could be detected in cardiovascular cells during mouse embryonic development and CBS mRNA was also detected in the aorta during late development^15^. In 2011, Sun, *et al.* detected the expression of CBS in the rat aorta and pulmonary artery rings^16^. The study by Kouichirou, *et al.* found that *in vitro*, L-cystathionine could significantly reduce superoxide radicals generated by human leukocytes in a concentration-dependent manner^17^. They also proved that L-cystathionine, through intraperitoneal injection, could significantly inhibit gastric erosions and lipid peroxidation caused by ischemia-reperfusion^18^. Maclean, *et al.* used mice and cell cultures to show that L-cystathionine could prevent fatty degeneration of the liver, acute tubular necrosis, and apoptosis caused by endoplasmic reticulum stress that was induced by tunicamycin^19^. We recently found that L-cystathionine could inhibit mitochondria-mediated macrophage apoptosis induced by ox-LDL via inhibition of cytc release and caspase activation^20^.

Inflammation is a non-specific immune response of the body to various traumatic stimulations, and its main function is to remove damaged tissue and invading pathogens, restoring the body to homeostasis. Throughout the whole formation and development process of atherosclerosis, inflammation plays an important role. Therefore, studying the regulatory mechanisms responsible for the inflammation occurring in a variety of vascular injury diseases, such as atherosclerosis, would be very important in the medical field. MCP-1 is an important cytokine in the regulation of the inflammatory response^21^. One study found that blocking MCP-1 could prevent early vascular inflammation^22^, and that it had an independent prognostic significance at the acute and chronic phase of acute coronary syndrome^23^. As an important transcription factor, NF-κB is a core part of the regulation of cellular inflammatory response. NF-κB normally exists in the form of a dimer, in which the most common form of a dimer is p65/p50. When NF-κB p65 is stimulated by a variety of signals, p65 phosphorylation is activated and transfers into the nucleus, thus initiating the transcription of target genes via binding to the corresponding sites and regulating the expression of related genes. Studies confirm that MCP-1 is one of the target genes of NF-κB regulation and that the promoter region of MCP-1 gene contains the binding sequence of NF-κB^24^.

The sulfur-containing amino acid metabolite H_2_S plays an important regulatory role in the occurrence of cardiovascular inflammation in human monocyte macrophages induced by ox-LDL^25^. Our previous study found that H_2_S blocked the activation of the NF-κB signaling pathway through inhibition of the phosphorylation of NF-κB, nuclear translocation of NF-κB, and DNA binding activity of NF-κB, which thereby inhibited the expression of MCP-1^26^. It was reported that H_2_S donors could attenuate an inflammatory response in atherosclerosis, while decreased endogenous H_2_S accelerated atherosclerosis^27^,^28^. Considering L-cystathionine was one of important precursors of endogenous H_2_S in the sulfur-containng amino acid metabolic pathway, in the present study we examined whether L-cystathionine had a regulatory effect on MCP-1 expression in the THP-1-derived macrophage via the NF-κB pathway, so as to provide useful information about the regulation of inflammation in a variety of diseases.

THP-1 cells can convert into macrophages which spread to the substrate with PMA treatment, while the cell line offers an important model in the study of the biological mechanism of macrophages^29^. The THP-1 cells were placed in serum-free RMPI 1640 nutrient solution for 24 hours before each experiment, for the purpose of synchronizing the cells. THP-1 cells were cultured *in vitro*, induced by PMA for 24 hours to differentiate into macrophages, and then were stimulated with 50 mg/L ox-LDL^30^. First, we studied the impact of L-cystathionine on the inflammatory cytokine MCP-1 secreted by THP-1-derived macrophages. The results showed that MCP-1 secreted by THP-1-derived macrophages under ox-LDL stimulation was increased but was inhibited by pretreatment with L-cystathionine. These results indicated that ox-LDL could induce the THP-1-derived macrophage inflammatory response (2-fold increase in MCP-1 expression) without any toxicity or induction of cell death at the dose used^30^. L-cystathionine could inhibit the inflammation (including a reduction by 50%) induced by ox-LDL, which suggests a very important biological protective significance of L-cystathionine in the inhibition of the inflammatory response.

Next, we explored the inhibitory mechanism of L-cystathionine on MCP-1 expression. The NF-κB p65 is the most important transcription factor in the regulation of cellular inflammatory response^31^,^32^. Therefore, we examined the possible mechanisms by which L-cystathionine inhibited inflammatory response via the NF-κB pathway. The results showed that phosphorylation of NF-κB p65 and its nuclear translocation in the THP-1-derived macrophage stimulated by ox-LDL were enhanced significantly, while those were reduced significantly after pretreatment with 0.3 mmol/L or 1.0 mmol/L L-cystathionine. NF-κ B p65 combining with κ B binding element in the promoter of NF-κB-dependent inflammatory cytokines (following the nuclear translocation of NF-κ B p65) is an important process in the regulation of inflammation. Therefore, we further studied the inhibitory effect of L-cystathionine on DNA binding activity of NF-κB p65. Our results suggest that ox-LDL stimulation could significantly increase DNA binding activity of NF-κB p65 in the THP-1-derived macrophage, while 0.3 mmol/L or 1.0 mmol/L L-cystathionine could significantly reduce the DNA binding activity.

Finally, we explored whether L-cystathionine could inhibit DNA binding level of NF-κB p65 with MCP-1 promoter in the THP-1-derived macrophage induced by ox-LDL using. ChIP method, a technique to study the interaction of *in vivo* protein and DNA. The results demonstrated that the DNA binding level of NF-κB p65 with MCP-1 promoter was increased significantly under ox-LDL stimulation, while 0.3 mmol/L or 1.0 mmol/L L-cystathionine could significantly reduce it.

However, the present study still has some limitations. Our research mainly focused on the NF-κB pathway in the exploration of the regulation of MCP-1. In fact, other signaling pathways, such as mitogen-activated protein kinase pathway, phospholipase C pathway, and Janus kinase-signal transduction and transcription activator pathway, were reported to play important roles in inflammatory regulation. It is necessary to further explore these pathways in the mechanistic studies of inflammatory regulation. In the present study, the analysis of the nuclear translocation of NF-κB p65 lacked quantitative evaluation, which was also a limitation to the study.

In summary, the study firstly discovered that L-cystathionine inhibited the THP-1-derived macrophage inflammatory response via inhibition of phosphorylation and nuclear translocation of NF-κB p65 and DNA binding with MCP-1 promoter induced by ox-LDL. These results are of great significance in the understanding of anti-inflammatory effects of L-cystathionine in inflammation-related diseases and might provide a novel target for prevention and treatment of inflammation-related diseases such as atherosclerosis.

## Materials and Methods

### THP-1 monocytes culture

THP-1 cells were purchased from ATCC Company, USA. The THP-1 cells with suspension growth were cultured in RMPI 1640 medium containing 10% fetal bovine serum, 100 U/mL penicillin and 100 U/mL streptomycin, which were placed in a constant temperature incubator containing 5% CO_2_ at 37 °C for culture. They were then differentiated into the adherent macrophages after 24 h of PMA (50 nmol/L) induction. The cells were placed in serum-free RMPI 1640 nutrient solution for 24 hours before each experiment to synchronize the cells^33^, and were incubated initially for 30 min before adding L-cystathionine (0.1 mmol/L, 0.3 mmol/L or 1.0 mmol/L, respectively) and then cultured for a certain time after adding ox-LDL (50 mg/L)^30^ (Peking Union-Biololgy Co. Ltd, Beijing, China). LDL was oxidized using 100 μM Cu_2_SO_4_ (oxidant) in PBS. Oxidation was terminated by adding excess EDTA. The protein percent of the product after oxidation was more than 97%, and concentration ranged from 1.0 mg/ml to 1.50 mg/ml.

### Analysis of MCP-1 expression levels in THP-1-derived macrophage supernatant by ELISA

Cell supernatant concentrations of MCP-1 were measured in duplicate by quantitative sandwich ELISA (R&D Systems, Minneapolis, MN, USA), according to the manufacturer’s instructions. A monoclonal antibody specific to MCP-1 was pre-coated onto a microplate. Standards and samples were pipetted into the wells and any MCP-1 present was bound by the immobilized antibody. After washing away any unbound substances, an enzyme-linked polyclonal antibody specific to MCP-1 was added to the wells. Following a wash to remove any unbound antibody-enzyme reagent, a substrate solution was added to the wells and color developed in proportion to the amount of MCP-1. The optical density of each well was determined within 30 minutes. The absorbance at 450 nm was taken as the ordinate and the concentration of standard substance was taken as the abscissa to draw the standard curve. The concentrations of MCP-1 in supernatant were calculated according to the absorbance of the samples.

### Expression of MCP-1 protein in THP-1-derived macrophage by western blotting

The ice-cold cell lysis buffer containing 50 mmol/L Tris-Cl (pH 7.4), 150 mmol/L NaCl, 1 mmol/L ethylenediamine tetra-acetic acid, 1% Nonidet (NP-40), 0.25% sodium deoxycholate, 1 mmol/L phenylmethylsulphonyl fluoride (PMSF), protease inhibitor and phosphatase inhibitor were used to extract the total protein of THP-1-derived macrophages. Protein concentration was measured by the Bradford method. Protein samples (30 μg) were separated in 10% sodium dodecyl sulfate polyacrylamide gel electrophoresis (SDS-PAGE) and then electrically transferred onto a polyvinylidene fluoride (PVDF) membrane. The membrane was blocked in skim milk for 1 hour and incubated with primary antibody against phosphorylated MCP-1 (Cell Signal Technology, Boston, MA, USA) at 4 °C for overnight, and then oscillatorily incubated with horseradish peroxidase (HRP)-conjugated secondary antibody at room temperature for 1 hour. Immunoreactions were visualized by electrochemical luminescence (ECL) and X-ray film (Eastman Kodak Company, Rochester, NY, USA) exposure.

### Phosphorylated NF-κB p65 protein in THP-1-derived macrophage by western blotting

The total protein of THP-1-derived macrophages was extracted and protein concentration was measured in accordance with the above methods and steps. Protein samples (20 μg) were separated in 10% SDS-PAGE and then electrically transferred onto the PVDF membrane. The membrane was blocked in skim milk for 1 hour and incubated with primary antibody against phosphorylated p65 (Cell Signal Technology, Boston, MA, USA) at 4 °C for overnight, and then oscillatorily incubated with horseradish peroxidase (HRP)-conjugated secondary antibody at room temperature for 1 hour. Immunoreactions were visualized by ECL and X-ray film (Eastman Kodak Company, Rochester, NY, USA) exposure. After exposure, the membrane was stripped with stripping solution for 30 minutes, blocked by milk, and incubated with primary antibody against NF-κB p65 at 4 °C overnight. It was then incubated with a secondary antibody and immunoreactions were visualized after exposure.

### Nuclear translocation of NF-κB p65 in the THP-1-derived macrophage by immunofluorescence assay

The THP-1-derived macrophages on slides were fixed in 4% paraformaldehyde at room temperature for 20 minutes and blocked with phosphate-buffered saline (PBS) containing 5% bovine serum albumin (BSA) for 30 minutes at 37 °C after washing with PBS. Then, the slides were incubated with primary antibody against phosphorylated p65 (1:50, diluted by antibody dilution) at 4 °C overnight. After washing with PBS, the slides were incubated with fluorescein isothiocyanate (FITC)-conjugated goat anti-rabbit IgG (1:50, diluted by antibody dilution) (Beijing Zhongshan Golden Bridge Biotechnology Company, China) in darkness at 37 °C for 1.5 hours. After washing with PBS, the slides were incubated with propidium iodide (Sigma, USA) in darkness for 5 minutes, and then mounted by an anti-fluorescence quencher (Beijing Zhongshan Golden Bridge Biotechnology Company, China) after washing with PBS. The slides were observed on a confocal fluorescent microscope, and green fluorescence indicated NF-κB p65 and red fluorescence indicated cell nucleus. Green and red overlapping in the cells represented NF-κB translocation.

### DNA binding activity of NF-κB p65 in the THP-1-derived macrophage by EMSA

Thermo nucleoprotein extraction kit (NE-PER Nuclear and Cytoplasmic Extraction Reagents) (Thermo Scientific, MA, USA) was used to extract nuclear protein. First, the cells were digested with pancreatin, and then the ice-cold CERI was added to precipitation after centrifugation (500 × g) at 4 °C for 5 minutes. The tubes were vortexed for 15 seconds, incubated on ice for 10 minutes, and ice-cold CERII was added. The tubes were then vortexed for 5 seconds and incubated on ice for 1 minute. The ice-cold NER was added to the precipitation section (nucleoprotein) to re-suspend after centrifugation (16,000 × g) at 4 °C for 5 minutes. The tubes were then vortexed for 15 sec × 4 times and kept on ice for 10 minutes each time, for a total of 40 minutes. The supernatant (nucleoprotein) was moved into a precooled tube for protein quantification (Bradford method) and stored at -80 °C after centrifugation (16,000 × g) at 4 °C for 10 minutes.

The second-generation EMSA kit of Roche labeled by digoxin (digoxigenin (DIG) gel shift kit) (Roche Applied Science, Mannheim, Germany) was used according to instructions. The brief steps were as follows: NF-κB consensus oligonucleotide (5′-AGTTGAGGGACTTTCCCAGGC-3′) was labeled by the DIG-ddUTP, and the labeled probe was incubated at room temperature for 15 minutes after mixing with 10 μg nuclear protein. The protein-DNA complexes were separated by 6% PAGE, electrically transferred onto a nylon membrane (Boehringer Mannheim Biochemica, Mannheim, Germany), and then the chemiluminescent band was detected.

### DNA binding activity of NF-κB p65 in the THP-1-derived macrophage by ELISA assay

The nucleoprotein of THP-1-derived macrophages was extracted and protein concentration was measured in accordance with the abovementioned methods and steps. Trans-AM NF-κB p65 Transcription Factor Assay kit (Active Motif, Carlsbad, CA, USA) was used to measure NF-κB p65 activity according to the instructions. The brief steps were as follows. The nucleoprotein (10 μg) was incubated in 96-well plates pre-coated with immobilized oligonucleotide containing a consensus binding site for p65 at room temperature for 1 hour. Primary antibody against NF-κB p65 was added to each well after washing by PBS, and then secondary antibody was added to each well after washing by PBS at room temperature for incubation for 1 hour. A substrate solution was added after washing again at room temperature for incubation for 5 minutes in the dark. The absorbance at 450 nm was measured after adding stop solution to terminate the reaction.

### Analysis of DNA binding level of NF-κB p65 with MCP-1 promoter in the THP-1-derived macrophage by ChIP

EpiQuikTM Chromatin Immunoprecipitation Kit (Epigentek, Farmingdale, NY, USA) was used to measure the DNA binding level of NF-κB p65 with MCP-1 promoter according to the instructions. The amplified sequences of MCP-1 primer were: forward, 5′-CCAGCCAAATGCATTCTCTTCTA-3′; reverse, 5′-GAGGTCAGTGCTGGCGTGA-3′. 1% paraformaldehyde was used to fix at room temperature for 10 minutes after washing the cells with PBS, and cell lysis buffer was used to scrape the cells after washing the cells with PBS again. Then, ultrasonic was used to shear chromatin (20–25% of the output power was used), 3-4 times for 10–12 seconds. The cells were placed on ice for 30-40 seconds between every 10–12 second session. If necessary, 5 μl ultrasonic cell lysis buffer was taken for agarose gel electrophoresis, and the length of the shear DNA fragments was usually 200–1000 bp. Lysates were cleared by centrifugation (16,000 × g) at 4 °C for 10 minutes, and the supernatant was taken. According to instructions, 10 μl of NF-κB p65 antibody was added to bond to the assay plate, and after the protein/DNA immunoprecipitation, reverse of crosslinked DNA, DNA purification and PCR amplification, the final PCR product was analyzed by 1.5% agarose gel electrophoresis.

### Statistical analysis

SPSS 16.0 software was used for statistical analysis. Data are expressed as 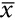 ± s, and ANOVA was used for mean comparison among groups. LSD test was used for further comparison between the two groups. P < 0.05 was considered statistically significant.

## Additional Information

**How to cite this article**: Zhu, M. *et al.* L-cystathionine inhibits oxidized low density lipoprotein-induced THP-1-derived macrophage inflammatory cytokine monocyte chemoattractant protein-1 generation via the NF-kB pathway. *Sci. Rep.* **5**, 10453; doi: 10.1038/srep10453 (2015).
